# The predictive power of pollination syndromes: Passerine pollination in heterantherous *Meriania macrophylla* (Benth.) Triana (Melastomataceae)

**DOI:** 10.1002/ece3.8140

**Published:** 2021-09-22

**Authors:** José Miguel Valverde‐Espinoza, Eduardo Chacón‐Madrigal, Olman Alvarado‐Rodríguez, Agnes S. Dellinger

**Affiliations:** ^1^ Escuela de Biología Universidad de Costa Rica San José Costa Rica; ^2^ Department of Botany and Biodiversity Research University of Vienna Vienna Austria; ^3^ Herbario Luis A. Fournier Origgi (USJ) Centro de Investigación en Biodiversidad y Ecología Tropical Universidad de Costa Rica San José Costa Rica; ^4^ Centro de Investigación en Estructuras Microscópicas Universidad de Costa Rica San José Costa Rica

**Keywords:** buzz pollination, division‐of‐labor hypothesis, heteranthery, melastomataceae, thraupidae

## Abstract

The cloud forest species *Meriania macrophylla* (Benth.) Triana has pseudocampanulate flowers with bulbous stamen appendages, typical for the passerine pollination syndrome found in the Melastomataceae tribe Merianieae. The species is further characterized by strong stamen dimorphism (heteranthery), a condition otherwise associated with pollen‐rewarding bee‐pollinated species (both in Melastomataceae and beyond). In passerine‐pollinated Merianieae, however, flowers usually only show weak stamen dimorphism. Here, we conducted field and laboratory investigations to determine the pollinators of *M. macrophylla* and assess the potential role of strong heteranthery in this species. Our field observations in Costa Rica confirmed syndrome predictions and indeed proved pollination by passerine birds in *M. macrophylla*. The large bulbous set of stamens functions as a food‐body reward to the pollinating birds, and as trigger for pollen release (bellows mechanism) as typical for the passerine syndrome in Merianieae. In contrast to other passerine‐pollinated Merianieae, the second set of stamens has seemingly lost its rewarding and pollination function, however. Our results demonstrate the utility of the pollination syndrome concept even in light of potentially misleading traits such as strong heteranthery.

## Graphical abstract

We tested the concept of pollination syndromes by investigating the tropical tree species *Meriania macrophylla*. In a previous study, this species had been predicted as passerine pollinated based on bulbous stamen appendages serving as nutritive food‐body reward for passerines. We confirm this prediction through field investigations and show that pollination syndromes are a useful concept if system specific floral functional traits are considered.

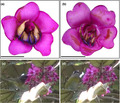

## Resumen

La especie de bosque nuboso *Meriania macrophylla* (Benth.) Triana presenta flores pseudocampanuladas con estambres con un apéndice bulboso, una morfología típica de las flores polinizadas por aves paserinas en Merianieae. Además, la especie se caracteriza por tener dos verticilos diferentes de estambres (heteroanteria), una condición que generalmente se asocia a especies polinizadas por abejas con polen como recompensa. En las especies de Merianieae polinizadas por aves la heteroantería es leve, si presente. Para este estudio determinamos los polinizadores de *M. macrophylla* y evaluamos posibles explicaciones de la presencia de estambres heteroantéricos Nuestras observaciones de campo en Costa Rica confimaron nuestras predicciones de que la polinización de esta especie se da por aves. Los conectivos engrosados funcionan como recompensa para las aves, que al removerlos de la flor activan el mecanismo de expulsión de polen. En contraste, el segundo verticilo de estambres aparentemente perdió tanto la función de recompensa como su función reproductiva. Nuestros resultados demuestran la utilidad de los síndromes de polinización incluso en presencia de caracteres confusos como la presencia de heteroanteria muy marcada.

## 1 INTRODUCTION

The idea that a plant's pollinator may be predicted from a plant's floral phenotype is central to the concept of pollination syndromes, which assumes recurring floral character combinations in adaptation to distinct pollinator groups (Dellinger, 2020; Faegri & van der Pijl, 1979; Fenster et al., 2004; Vogel, 2012). The extent to which pollination syndromes are reliable predictors of a plant's pollinator is under debate, however (Abrahamczyk et al., 2017; Dellinger, 2020; Ollerton et al., 2009). A considerable mismatch between predicted and observed pollinators has been reported when traditional, angiosperm‐wide syndromes, usually based on few, relatively crude, categorical traits, were used for predictions (Abrahamczyk et al., 2017; Ollerton et al., 2009; but also see Ashworth et al., 2015; Johnson & Wester, 2017; Rosas‐Guerrero et al., 2014). When using more refined, system‐specific and objective (e.g., quantitative) trait datasets, the predictive accuracy was generally higher (Abrahamczyk et al., 2017; Armbruster et al., 2011; Dellinger, Artuso, et al., 2019; Dellinger, Chartier, et al., 2019).

While much debate in recent years has focused on whether or not pollination syndromes are reliable tools to predicting pollinators, little attention has been given to the factors and floral traits generating prediction inaccuracy (Dellinger, 2020). Prediction inaccuracy may be associated with the methods and traits used to predict pollinators, fluctuations in pollinator communities, trade‐offs arising from interactions with floral antagonists, or evolutionary (i.e., parallel adaptation to current and ancestral pollinators), genetic, and developmental constraints inherent to the taxa under study (e.g., Ashworth et al., 2015; Caruso et al., 2018; Dellinger, Artuso, et al., 2019; Johnson & Wester, 2017). Particularly in flowers with functionally and structurally complex pollination mechanisms, developmental constraints may be strong and hinder convergence into distinct syndromes in all traits (Armbruster, 2002; Dellinger, Artuso, et al., 2019). In order to guarantee high predictive accuracy in such systems, traits important in differentiating syndromes have to be identified and considered independently of uninformative traits. Objective statistical classification methods such as machine‐learning algorithms have been proposed as useful in identifying such traits (Dellinger, Chartier, et al., 2019; Johnson, 2013). These algorithms are first trained on floral trait datasets of species with empirically observed pollinators, and model accuracy is validated by assessing whether models can indeed correctly predict pollinators of these species. If predictions are accurate, these algorithms may then be used to predict pollinators for species currently lacking empirical observations (see Johnson, 2013 for detailed explanation). Empirical verification of predictions stemming from statistical classification algorithms remains scarce, however (Dellinger, Scheer, et al., 2019; Lagomarsino & Muchhala, 2019).

In the Neotropical plant tribe Merianieae (ca. 300 species, Melastomataceae), system‐specific pollination syndromes have been described recently using statistical classification methods (Random Forest analyses, Dellinger, Chartier, et al., 2019). A morphologically diverse bee–buzz pollination syndrome was found to be most common, with repeated independent transitions into a passerine syndrome and a mixed‐vertebrate pollination syndrome (Dellinger, Chartier, et al., 2019). The passerine pollination syndrome of Merianieae is characterized by pseudocampanulate corollas and bulbous stamen appendages, which function in attracting passerine birds and in expelling pollen through an explosive bellows mechanism triggered when a foraging passerine grabs an appendage with its bill (Dellinger et al., 2014). Further, the bulbous stamens function in rewarding the birds since they contain high percentages of hexose sugars (Dellinger et al., 2014).

**FIGURE 1:**
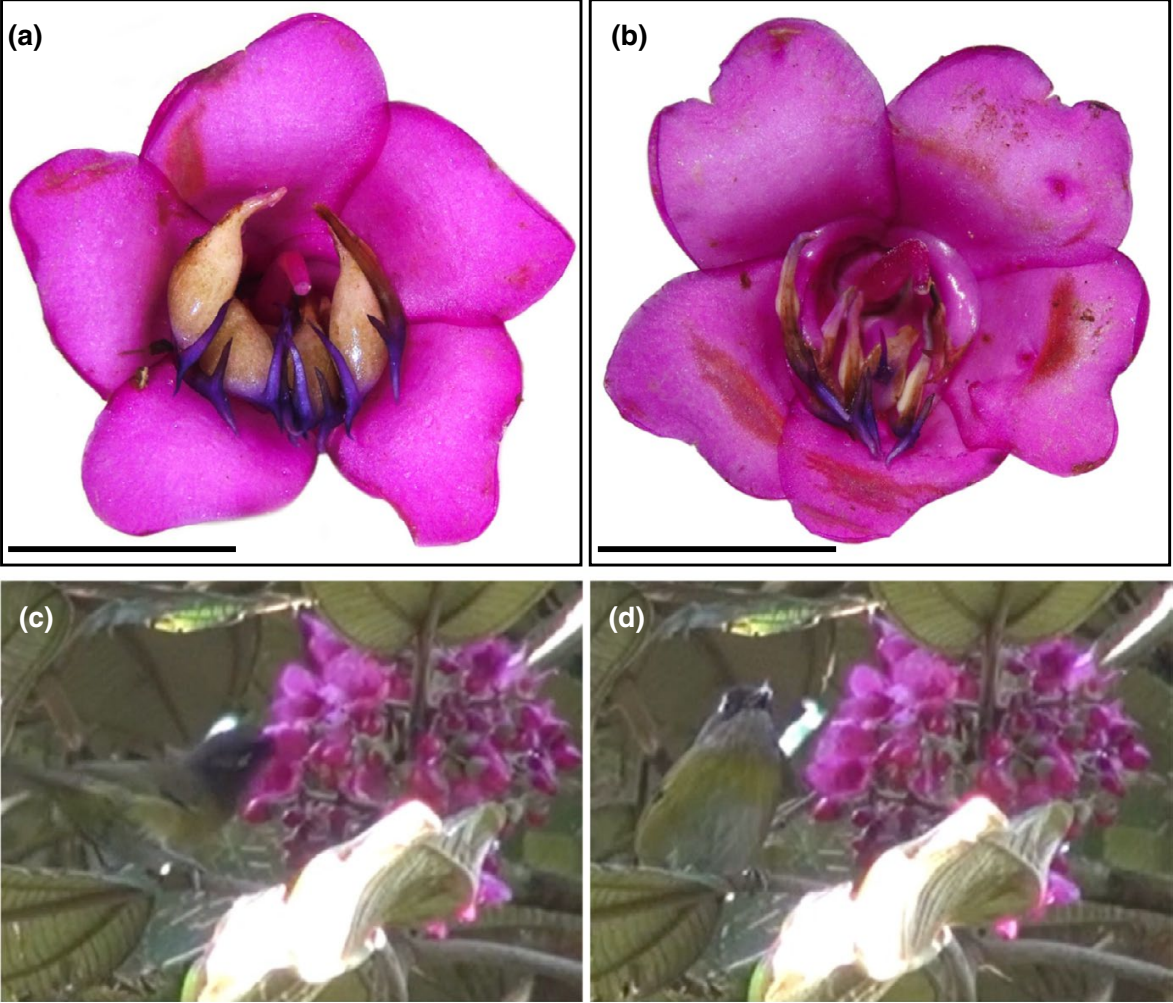
(a) Fresh flower of *Meriania macrophylla*, (b) flower after removal of thick stamens by birds, and (c, d) *Chlorospingus ophthalmicus* removing stamens from the flowers of *Meriania macrophylla*

Our study species, *Meriania macrophylla* (Benth.) Triana, was predicted as passerine pollinated by statistical classification methods (Dellinger, Chartier, et al., 2019) since it shares these most distinguishing traits with passerine‐pollinated Merianieae. *Meriania macrophylla* differs, however, from Merianieae species with documented passerine pollinators in having strongly dimorphic stamens with bifurcated secondary appendages (Figure 1). Such strong stamen dimorphism (i.e., in color, shape, and size) and complex elongated appendages have commonly been associated with pollen‐rewarding, bee‐ and buzz‐pollinated flowers (i.e., flowers where pollen is released through vibrations applied by bees; Bochorny et al., 2021; Vallejo‐Marín et al., 2010). In these flowers, the evolution of heteranthery is explained by the dual and conflicting function of pollen as reward and reproductive agent (Vallejo‐Marín et al., 2010). To alleviate this “pollen dilemma,” heteranthery is usually believed to function in “division of labor,” with the large, conspicuous stamen‐type functioning in pollinator rewarding, and the small, inconspicuous stamen‐type functioning in pollen transfer (Luo et al., 2008; Vallejo‐Marín et al., 2009). In Merianieae, strong stamen dimorphism also mostly occurs in pollen‐rewarding bee‐ and buzz‐pollinated species (Dellinger et al., 2021). Recent investigations have shown, however, that heteranthery is not restricted to species with pollen rewards (and hence does not constitute a helpful trait in differentiating syndromes in this group), but that weak heteranthery is common also in passerine‐pollinated Merianieae (Dellinger et al., 2021). In these species, heteranthery functions in staggered pollen release, with foraging passerines first removing the bigger stamen type and only later in anthesis, on separate visits, removing the small stamen type. To date, it remains unclear whether the marked heteranthery in *Meriania macrophylla* also functions in staggered pollen release with passerine birds, or, alternatively, indicates parallel adaptations to bee pollinators (the ancestral pollinators in the group; Dellinger, Chartier, et al., 2019).

In this study, we employ empirical field observations to validate the predictive accuracy of pollination syndromes based on statistical classification algorithms. Specifically, we test whether traits identified as “most discriminating” (bulbous connectives, food‐body reward, urceolate corolla) by classification algorithms (Dellinger, Chartier, et al., 2019) are indeed reliable in predicting the correct primary (passerine) pollinators. Further, we ask whether traits detected as uninformative in differentiating syndromes (heteranthery) are indicators of secondary (ancestral bee) pollinators? We use morphological, functional, and calorimetric assessments of stamens to investigate adaptations to either passerine or bee pollination and to fully describe a highly unusual case of heteranthery, unrelated to the division‐of‐labor hypothesis.

## 2 METHODS

### 2.1 Study species

*Meriania macrophylla* is a tree of 6–21 m, distributed from Mexico to Guatemala and from Costa Rica to Venezuela in tropical forests between 1,400 and 2,600 m a.s.l. (Almeda, 1993; Calderón‐Sáenz & Mendoza‐Cifuentes, 2000). It is a rare species throughout its distribution, with few known low‐density populations, and probably one of the most endangered species in the family (Almeda, 1993, 2007). Flowers (ca. 1.7 cm in diameter) are hermaphroditic and appear in terminal panicles. The flowers are pentamerous and actinomorphic, with magenta petals and two sets of stamens: antepetalous stamens with a bulbous white connective and antesepalous stamens with a flat white connective (thick and thin stamens, respectively, hereafter). All stamens bear slender, bifurcated violet appendages. The androecium is zygomorphically arranged, and the style arches over the androecium (Almeda, 1993, Figure 1).

### 2.2 Study site and pollinator observations

We conducted fieldwork in Vara Blanca, Heredia Province, Costa Rica (10°09′N, 84°09′W, 1,860 m a.s.l.), 11–16 October and 2–4 November 2018. The study population was located in montane forest remnants between pastures used for livestock grazing. We conducted pollinator observations continuously from 5:00–15:00 without breaks. Each day, we selected one tree with abundant flowers to observe using binoculars (90 direct observation hours). We noted down each animal that we observed interacting with anthetic flowers. In addition, we used four video cameras (Sony HDR‐CX700) to monitor single inflorescences with anthetic flowers in the same tree that was being observed. The video cameras were installed at least five meters away from the inflorescences and could film for two 4‐hr periods each day (105.6 camera observation hours). We later replayed the videos on a laptop to identify pollinators and record the number of flowers visited and the visit duration. We considered a visitor as a pollinator if it contacted the reproductive organs and triggered pollen release. Since we only observed pollen release through stamen removal (bellows mechanism), we only considered visits with stamen removal as effective. For bird visitors capable of removing stamens, we further noted down the number of stamens they removed (Dellinger et al., 2014). We identified bird visitors to species using the app Merlin Bird ID (Cornell University, 2018).

Another population was located in Aserrí, San José Province (09°42′N, 84°06′W, 2,164 m a.s.l.), but because of the trees' height, this population was not used for pollination observations.

### 2.3 Stamen morphology and function

We collected fresh flower material in FAA (formaldehyde, alcohol, and acetic acid; for a better preservation of the plant tissue), from both Vara Blanca and Aserrí populations, for morphological analyses. After a week, we transferred flowers to 75% ethanol to prepare the samples for analysis in *SEM* (scanning electron microscopy).

To quantitatively compare the two stamen types, we photographed 45 stamens of each type under an Olympus SZX16 stereoscope and used the software ImageJ to measure the length and width of the stamens (Schneider et al., 2012). We measured the length as the diagonal between the apex of the anther and the beginning of the appendage, and the width in the widest part of the connective (Figure S1). We performed a Wilcoxon rank‐sum test, (non‐normal distribution of the data), using RStudio (R Core Team, 2016; RStudio Team, 2020) to test for significant differences in stamen size between stamen types. We report means and standard deviations of stamen sizes.

To assess potential structural differences between the two stamen types, we prepared three stamens of each type for *SEM*. We washed the stamens with 0.2 M phosphate buffer for 15 min each, then did postfixation with 2% osmium tetroxide (OsO_4_) for 2 hr, followed by five washes with distilled water, 10 min each.

To study internal stamen structures, we used the cryofracturing technique following the protocol by Tánaka (1989). We treated stamens with dimethyl sulfoxide (DMSO) at concentrations of 25% and 50%, 30 min each, then froze them in a metal plate with liquid nitrogen, and gently broken anthers with a frozen hammer and scalpel. We treated the broken and frozen tissue with DMSO at 50% and 25% to defrost and then washed stamens five times with distilled water, 10 min each. We fractured thick stamens only using the scalpel.

We dehydrated all samples over an ethanol series (increasing concentrations: 30%, 50%, 70%, 80%, 90%, 95%, and two baths of 100%). We then put stamens in an isoamyl acetate (C_7_H_14_O_2_)–ethanol bath (1:1, 15 min), followed by a 100% isoamyl acetate bath. We used a Leica EM CPD300 critical point dryer (Leica Mikrosysteme GmbH, Austria) to critical point dry stamens. We then mounted the samples on 50‐mm plates and covered them with gold using a Quorum EMS 150RS coater (Quorum Technologies Ltd, UK). We photographed samples using a Hitachi 3700N scanning electron microscope (*SEM*) in the laboratory in the CIEMIC (Centro de Investigación en Estructuras Microscópicas, Universidad de Costa Rica).

In order to understand whether *M. macrophylla* is adapted to bird pollination through the bellows mechanism (Dellinger et al., 2014), we used forceps to compress the connectives, mimicking a bird's bill. We did this test in 10 freshly collected flowers for all the ten stamens in flowers from the two populations available.

### 2.4 Pollen amount and morphology

Differences in pollen amount, pollen grain features, and viability have been reported for heterantherous species (Pinheiro‐Costa et al., 2018). To assess potential differences in pollen amount, we prepared three single stamens of each stamen type for pollen counting following the method of Dellinger, Scheer, et al. (2019). We placed single stamens into Eppendorf tubes filled with 1,000 µl purified water and squeezed them with a pestle to extract all pollen grains. We then placed the tubes into a sonication bath for 15 min to remove potential residual pollen grains. We injected 100 µl of the pollen solution into a multichannel particle counter (Topas Particle Counter FAS362B). We only selected the size classes around the pollen grain sizes we measured under *SEM* (see below) to calculate the average pollen amount per stamen type.

We used *SEM* to compare the morphology and size of pollen grains from the two stamen types. We recorded pollen grain polarity, presentation, scope, and type and number of apertures. We described the shape following Erdtman (1969) using the polar axis and equatorial diameter ratio of 10 pollen grains. We measured pollen grain diameter exclusively in the equatorial view to avoid bias. We used Student's *t* test to compare pollen sizes between the two stamen types and report means and standard deviations.

### 2.5 Calorimetric measurements of stamens

In passerine‐pollinated Merianieae, the bulbous connectives function as nutritive food‐body rewards (Dellinger et al., 2014). We used calorimetric analyses to assess the nutritive value of the two stamen types of *M. macrophylla*. We removed single stamens from flowers, separated them into either of the two types, and dried them for 2 min in a microwave oven at highest energy (Dellinger et al., 2014). We then prepared four different samples for calorimetric measurements: two samples containing either entire thick or entire thin stamens, and two samples containing only the connectives of either thick or thin stamens. For the latter two, we removed the connectives from the rest of the stamens using a scalpel. We pulverized each of the four samples and then compressed the powder into a small pellet. We measured the calorimetric content of each pellet separately using an IKA calorimeter C 2000 basic Version 1 (IKA®‐Werke GmbH & Co. KG, Germany) at the Department of Nutritional Sciences, University of Vienna.

## 3 RESULTS

### 3.1 Empirical validation of pollination syndrome prediction

Objective statistical classification algorithms had predicted passerine bird pollination for *M. macrophylla* given its typical bulbous stamen connectives (Dellinger, Chartier, et al., 2019). We observed flies, bumblebees, wasps, lepidopterans, and birds as floral visitors. Conforming to syndrome prediction, however, only passerine birds were frequent visitors and could activate the bellows pollen expulsion mechanism by removing stamens from flowers (Video S1). We did not observe any of the occasionally visiting insects to extract pollen or touch the reproductive organs. Flies and lepidopterans may use flowers for oviposition, given the high number of larvae found in stamens and gynoecia.

**FIGURE 2:**
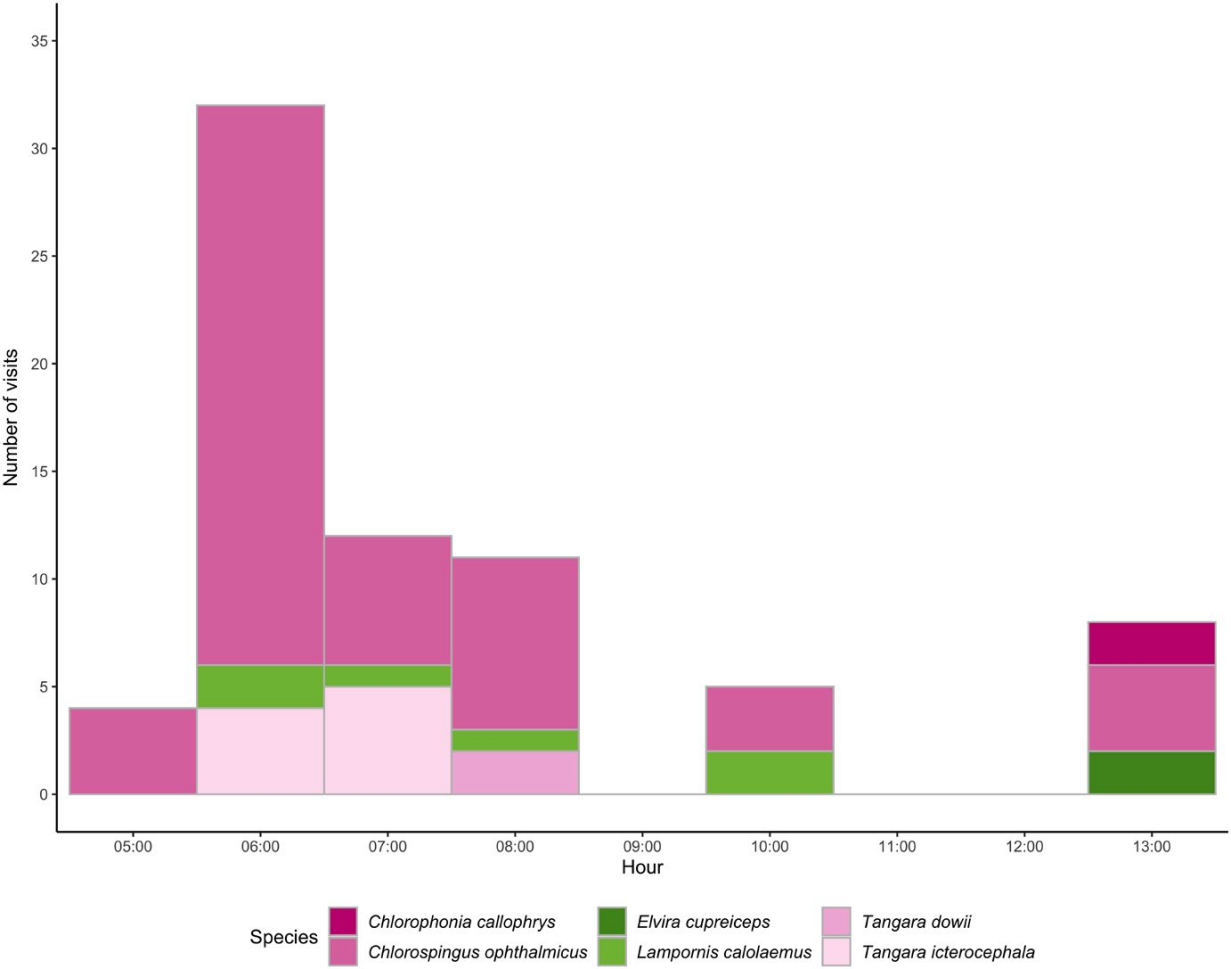
Number of visits by hour of the six bird species observed to approach the flowers of *Meriania macrophylla,* pollinating passerine birds are shown in pink, and nonpollinating hummingbirds in green

Overall, we observed six bird species, four passerine birds, and two hummingbirds (Table 1). The common bush tanager, *Chlorospingus ophthalmicus,* was most abundant with 51 effective visits during the observation period (Figures 1c,d and 2). The silver‐throated tanager, *Tangara icterocephala,* was a recurrent visitor, while the golden‐browed chlorophonia, *Chlorophonia callophrys*, and the spangle‐cheeked tanager, *T. dowii,* only visited a few flowers on a single day (Figure 2). Two hummingbird species (Trochilidae) were observed approaching the flowers but neither removed stamens nor triggered pollen release. Overall, bird visitation was highest in the morning hours between 06:00 and 08:00, no visits were observed after 13:00.

**TABLE 1:** Bird species that visited the flowers of *Meriania macrophylla*

Order	Family	Species	Stamen removal
Apodiformes	Trochilidae	*Elvira cupreiceps* (Lawrence, 1867)	No
		*Lampornis calolaemus* (Salvin, 1865)	No
Passeriformes	Fringillidae	*Chlorophonia callophrys* (Cabanis, 1861)	Yes
	Thraupidae	*Chlorospingus ophtalmicus* (De Bus de Gisignies, 1847)	Yes
		*Tangara dowii* (Salvin, 1863)	Yes
		*Tangara icterocephala* (Bonaparte, 1851)	Yes

### 3.2 Stamen removal and pollen expulsion

All passerine birds behaved similarly when visiting flowers (Video S1). They perched in front of inflorescences and visited multiple inflorescences and flowers during each foraging bout. When foraging, the bird introduced part of its head into the flower to rip out a thick stamen by the bulbous connective with its beak and meanwhile touched the exerted stigma. The bird chewed the stamen and then spit it out. The pressure produced by the compression of the bulbous connective by the beak resulted in the expulsion of a cloud of pollen and liquid (potentially phloem sap), from the stamen. This pollen landed on the bird's face and was transferred to the stigma when the bird removed another stamen. The birds never removed thin stamens; those remained in the flowers and withered (Figure 1b). This pattern was observed in flowers from the two populations studied. The most frequent visitor, *Chlorophonia ophthalmicus,* removed 1.31 (±1.52) stamens per visited flower on average.

### 3.3 Stamen morphology and function

**FIGURE 3:**
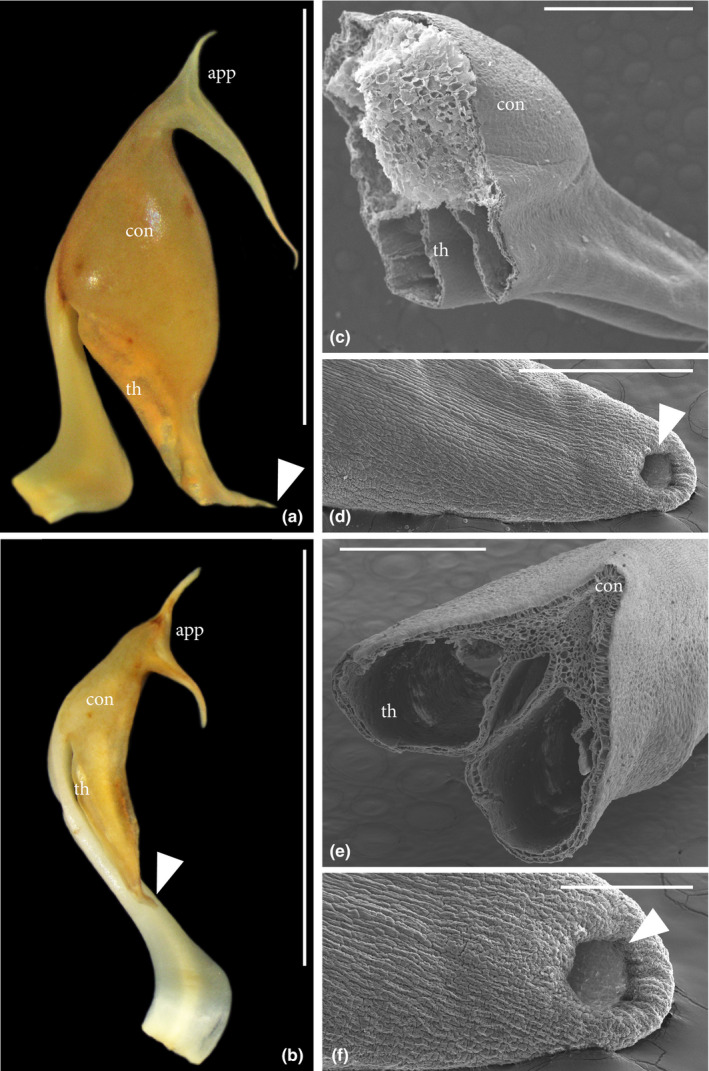
Details of the stamens of *Meriania macrophylla* under light (a, b) and electron microscopy (c–f). Whole thick stamen (a), whole thin stamen (b), cross section (c) and apical pore (d) of a thick stamen, and cross section (e) and apical pore (f) of a thin stamen. app, appendage; con, connective; th, thecae

The flowers of *M. macrophylla* are strongly heterantherous, a trait commonly associated with buzz pollination by bees. The stamen types differ in color, size, and shape of their connectives: Thick stamens bear large, bulbous white connectives, while connectives are barely enlarged and dirty whitish in small stamens (Figures 1a,b and 3). In addition, both stamen types bear prominent, slender, bifurcated appendages. Histologically, the connective appendages are composed of densely arranged parenchyma with a prominent vascular bundle. Structurally, the only difference between the two stamen types lies in the greater volume of the connective parenchyma of the thick stamens. Thecal walls of both stamen types are smooth, and septa between pollen chambers have collapsed so that each theca only consists of one pollen chamber. The thecae merge at the apex where a single pore is located (Figure 3). The thick stamens have significantly longer (9.1 mm, ±1.3) and wider (3.6 mm, ±0.5) thecae than the thin stamens (length 8.1 mm, ±1.3; width 1.6 mm, ±0.3; length *W* = 533.5, *p* < .001, width *W* = 3, *p* < .001).

When testing for pollen expulsion through the bellows mechanism by artificially compressing connectives using forceps, we found pollen release only from the thick stamens (Video S2). In addition to pollen, a clear liquid was extruded from the connective tissue.

### 3.4 Pollen amount and morphology

Thick stamens produced almost twice as much pollen as small stamens (49,240 ± 5,798 vs. 29,270 ± 18,019). Pollen did not differ in external structures: In both stamen types, the pollen is an isopolar monad with a spherical contour and six colpi (Figure S2). The grain has an oblate‐spheroidal shape, based on a mean ratio of 0.98 (± 0.06) μm. Pollen grains did not differ in shape between the two stamen types. Pollen grains of thick stamens were significantly larger (12.35 µm ± 0.52) than pollen grains of thin stamens (12.08 µm ± 0.61, *t* = −2.14, *df* = 82.19, *p* < .05).

### 3.5 Caloric content of stamens

The thick stamens are more than twice as nutritious as the thin stamens (Table 2). Both weight and caloric contents of the thin stamens' connectives are so low that we could not assess the caloric content of the thin connectives separately (Table 2). The total caloric content of a flower is around 460.8 J. Since passerine birds only consumed the thick stamens, the maximum energetic input is 328 J/flower (0.08 kcal/flower). With approximately 1.3 stamens removed per visit, the mean energetic intake per visit of *C*. *ophthalmicus* was around 86.07 J.

**TABLE 2:** Caloric content of both types of stamens of *Meriania macrophylla*

Stamen	Sample	Weight of the sample (g)	Weight of one stamen (g)	Energy (J/g)	Energy (J/stamen)
Thick	Whole	1.21690	0.0043	15,279	65.7
	Appendage	0.12850	0.0020	16,136	35.49
Thin	Whole	0.19910	0.0015	17,642	26.46
	Appendage	–	–	–	–

## 4 DISCUSSION

In agreement with statistical pollination syndrome predictions, *Meriania macrophylla's* primary pollinators are passerine birds of the family Thraupidae. Although strong heteranthery and bifurcated stamen appendages could also indicate bee pollination, we did not find bees or any other functional group as secondary pollinators. Our results demonstrate the value of objective classification methods for pollinator predictions since machine‐learning algorithms correctly identified traits important in circumscribing the passerine syndrome, such as the multifunctional bulbous stamen connectives, and traits uninformative in delineating syndromes in Merianieae (such as heteranthery).

*Meriania macrophylla* was visited by a broad set of different functional pollinator groups (Fenster et al., 2004), but only passerine birds triggered pollen release. While the bulbous stamen connectives indicated passerine birds as pollinators, we did initially not rule out bees as potential secondary pollinators. Bee–buzz pollination is ancestral in Merianieae (Dellinger, Chartier, et al., 2019), and ancestral pollinators are often retained as secondary pollinators (Rosas‐Guerrero et al., 2014). None of the occasionally visiting bees was observed buzzing the flowers to extract pollen, however. Hummingbird pollination does also occur in Merianieae, but is, again, associated with a different pollen‐release mechanism (salt‐shaker‐like pollen release from pendant flowers) and nectar rewards. In Merianieae, nectar is secreted from the stamens, either through clearly visible ruptures on the filament or connective joint or through porous tissue on the filament (Dellinger, Scheer, et al., 2019). Neither did we find such structures when assessing stamens under the *SEM* nor did we find any nectar when assessing fresh flowers in the field. While hummingbirds commonly visit flowers for nectar rewards, it is also possible that hummingbirds visit flowers in search of small insects to eat (Young, 1971). Alternatively, hummingbirds may have looked after potential leftovers from passerine feeding: We observed the extrusion of a liquid (possibly phloem sap) when passerine birds chewed the removed stamens. The passerines spit out the stamens again after chewing (the amount of liquid ingested from each chew is unknown); some of this liquid may spill on petals from where it could potentially be taken up by hummingbirds. Even if this highly unlikely scenario applies, however, hummingbirds are not capable of activating the bellows mechanism (Dellinger et al., 2014) and hence do not serve as pollinators in *M. macrophylla*.

As typical for the passerine syndrome, stamens in *Meriania macrophylla* serve as multifunctional organs in pollinator attraction, rewarding, and pollen release (Dellinger et al., 2014). With 15.279 J/g, stamens of *M. macrophylla* are highly nutritious, exceeding the caloric value of most fruits and of bulbous stamens of other passerine‐pollinated Merianieae (*Axinaea*, Dellinger et al., 2014; Schaefer et al., 2003; Vinson et al., 2005). *M*. *macrophylla's* stamen connectives differ structurally from those known of other passerine‐pollinated species of the genus *Axinaea:* While the bulbous connective tissue is aerenchymatic in *Axinaea* (Dellinger et al., 2014), the bulbous connectives of *M. macrophylla* consist of relatively dense parenchymatic tissue. Also, the compression of the connectives leads to the release of a clear liquid in *M. macrophylla*, but not in *Axinaea*. Overall, however, the bellows mechanism in *M. macrophylla* follows the same functional principle as in *Axinaea*, with pollen release only affected by a forceful compression of the bulbous connective. This contrasts to other pneumatic pollen‐release mechanisms described for bee‐pollinated plants such as *Cyphomandra* (Solanaceae; Sazima et al., 1993). In *Cyphomandra*, fragrance‐collecting bees push against the soft thecal walls and may thereby cause pollen release (Sazima et al., 1993). Thecal walls are smooth and sturdy in *M. macrophylla*, as typical for the passerine syndrome, and no pollen can be released when pushing against the thecae.

Flowers of *M. macrophylla* show traits otherwise typical for the bee–buzz pollination syndrome, albeit of minor importance in differentiating syndromes in Merianieae, such as heteranthery or conspicuous bifurcated connective appendages (Figure 3, Dellinger, Chartier, et al., 2019). Since passerine pollination evolved from bee pollination in Merianieae (Dellinger, Chartier, et al., 2019), the presence of these traits may partially reflect the species' evolutionary background rather than adaptations to its present‐day pollinators (Li & Huang, 2009; Rosas‐Guerrero et al., 2014). In bee‐ and buzz‐pollinated Merianieae, stamens usually bear conspicuously enlarged, rigid appendages composed of parenchymatic tissue. These appendages serve as handles for bees to grab when applying vibration buzzes to extract pollen (Dellinger, Chartier, et al., 2019). The slender bifurcated appendages borne by both stamen types in *M. macrophylla* may be residual handles for bee pollination. We did not observe any function of these appendages in the bellows mechanism, however.

The strong heteranthery observed in flowers of *M. macrophylla*, on the contrary, likely evolved de novo with the shift to passerine pollination (Dellinger et al., 2021). Recent macroevolutionary analyses across Merianieae have demonstrated a surprising association between heteranthery and shifts to food‐body rewarding and passerine pollination (Dellinger et al., 2021). While the evolution and function of heteranthery are commonly explained by a plant's need to reconcile the pollen dilemma through “division of labor” in bee‐ and buzz‐pollinated flowers (Barrett, 2002; Vallejo‐Marín et al., 2010), this clearly does not satisfactorily explain its function in food‐body rewarding, passerine‐pollinated species. Instead, Dellinger et al. (2021) showed that in the Merianieae genus *Axinaea*, passerines first remove the large (outer) stamen whorl and only later in anthesis remove the small (inner) stamen whorl. The large stamens are twice as nutritious as the small stamens, but stamens ripen in the course of anthesis so that, at the end of anthesis, small stamens are equally nutritious as large stamens were early in anthesis (Dellinger et al., 2021). The authors interpreted heteranthery as pollen dosing strategy to assure multiple independent pollinator visits to the flowers. Rewarding pollinators with stamens (food bodies) is particularly risky (Simpson & Neff, 1981): If all stamens were consumed at the first visit, each flower would disperse its pollen only to one pollen vector, reducing male fitness (Kay et al., 2020). The story seems somewhat different in *M. macrophylla*, however. Our observations showed that only the thick stamen type is functional in pollinator rewarding and pollen transfer. Given that thin stamens are barely visible in freshly anthetic flowers, they may not even contribute to pollinator attraction. Our observations indicate that thin stamens usually remain in flowers (Figure 1b) and may have completely lost their function in the pollination process, which is surprising since they still produce (energetically costly and likely fertile) pollen. Further, passerines usually remove all five thick stamens in one visit, putting *M. macrophylla* flowers at exactly the risk mentioned above.

If heteranthery does neither function in “division of labor” nor in pollen dosing, why did it evolve de novo in *M. macrophylla*? We believe that heteranthery may be the result of a developmental‐spatial constraint in these flowers (Dellinger et al., 2014). Flowers of *M. macrophylla* are relatively small and appear densely filled by the five thick stamens (Figure 1a). With the evolution of food‐body rewards and the enlargement of the connectives, there may simply not have been enough space for ten bulbous stamens within a flower. In the approximately 40 *Axinaea* species, on the contrary, corollas spread more at anthesis and the two stamen whorls differ in filament length so that ten moderately heterantherous stamens may be accommodated spatially (Dellinger et al., 2021).

Finally, strong heteranthery with bulbous stamen connectives characterizes the entire clade (ca. 6 (sub)species) *Meriania macrophylla* belongs to (e.g., *Meriania franciscana* C. Ulloa & Homeier*, Meriania peltata* L. Uribe). During field investigations in Colombia, we found flowers of *M. peltata* to also have only thick stamens removed, with thin stamens remaining (Dellinger, pers. obsv.) and a yellow‐eared parrot has recently been observed as pollinator of *M. peltata* (feeding on the stamens and thereby activating the bellows mechanism) in the Páramo de Anaime (Colombia, Departamento del Tolima; Diego Fernando Espitia & Mauricio Posada, pers. com.). It is hence highly plausible that the entire clade is pollinated by birds capable of activating the bellows mechanism. Further studies are needed, however, to investigate possible differences in the functional significance of heteranthery between *Axinaea* species and the *M. macrophylla* group.

## CONFLICT OF INTEREST

None declared.

## AUTHOR CONTRIBUTIONS

**José Miguel Valverde‐Espinoza:** Conceptualization (equal); data curation (equal); formal analysis (equal); investigation (equal); methodology (equal); project administration (equal); resources (equal); visualization (equal); writing–original draft (equal); writing–review and editing (equal). **Eduardo Chacón‐Madrigal:** Methodology (equal); resources (equal); supervision (equal); writing–review and editing (equal). **Olman Alvarado‐Rodríguez:** Methodology (equal); resources (equal); writing–review and editing (equal). **Agnes S. Dellinger:** Conceptualization (equal); methodology (equal); resources (equal); supervision (equal); validation (equal); writing–review and editing (equal).

## Supporting information

Fig S1‐2

Video S1

Video S2

## Data Availability

Morphological data of pollen and stamens, and bird visitation data are uploaded in Dryad, as well as the R script for making the graphs and the descriptive statistics (Valverde‐Espinoza, Jose Miguel (2021), Meriania macrophylla_field data, Dryad, Dataset, https://doi.org/10.5061/dryad.sqv9s4n4s).
