# Supplementary material for: The predictive power of pollination syndromes: Passerine pollination in heterantherous Meriania macrophylla (Benth.) Triana (Melastomataceae)
Source: Ecol Evol. 2021 Sep 22;11(20):13668–77. doi: 10.1002/ece3.8140 (PMC8525179; doi:10.1002/ece3.8140)
Supplement: Supplementary file 1 — Fig S1‐2 [file ECE3-11-13668-s003.docx]

**The predictive power of pollination syndromes: passerine-pollination in heterantherous *Meriania macrophylla* (Benth.) Triana**

**Supplementary Files**

**Figure S1:** Measurements made to the thin (left) and thick (right) stamens of *M. macrophylla*. L: length, W: width.

**Figure S2:** SEM images of a whole grain of pollen from the thin (A) and thick (B) stamens. There was no difference in pollen grain size, shape or ornamentation. We hence believe that pollen grains from both thin and thick stamens are fertile.

**Video S1:** Visitation and stamen removal by *Chlorospingus ophtalmicus* in flowers of *Meriania macrophylla*, Vara Blanca, Heredia, Costa Rica.

**Video S2:** Pollen expulsion, in a thick stamen of *Meriania macrophylla*, using forceps to compress the connectives. It can be seen the besides pollen there’s a liquid component expelled from the stamens.
